# Feasibility and safety of high-dose adenosine perfusion cardiovascular magnetic resonance imaging

**DOI:** 10.1186/1532-429X-13-S1-P105

**Published:** 2011-02-02

**Authors:** Theodoros D Karamitsos, Ntobeko BA Ntusi, Jane M Francis, Cameron J Holloway, Saul G Myerson, Stefan Neubauer

**Affiliations:** 1 Oxford Centre for Clinical Magnetic Resonance Research, OXFORD, UK

## Objective

The aim of the present study was to assess the tolerance and safety of a high-dose adenosine protocol (210mcg/kg/min) in patients with inadequate hemodynamic response to the standard adenosine dose (140mcg/kg/min) when undergoing CMR perfusion imaging.

## Introduction

Adenosine is the most widely used vasodilator stress agent for Cardiovascular Magnetic Resonance (CMR) perfusion studies. With the standard dose of 140 mcg/kg/min some patients fail to demonstrate characteristic hemodynamic changes: a significant increase in heart rate (HR) and mild decrease in systolic blood pressure (SBP). Whether an increase in the rate of adenosine infusion would improve peripheral and, likely, coronary vasodilatation in those patients is unknown.

## Methods

98 consecutive patients with known or suspected coronary artery disease (CAD) underwent CMR perfusion imaging at 1.5 Tesla. Subjects were screened for contraindications to adenosine, and an electrocardiogram was performed prior to the scan. All patients initially received the standard adenosine protocol (140 mcg/kg/min for at least 3 minutes). If the hemodynamic response was inadequate (HR increase <10bpm or SBP decrease <10mmHg) then the infusion rate was increased up to a maximum of 210 mcg/kg/min (maximal infusion duration 7 minutes).

## Results

All patients successfully completed the CMR scan. Of a total of 98 patients, 18 (18%) did not demonstrate evidence of a significant increase in HR or decrease in SBP under the standard adenosine infusion rate. Following the increase in the rate of infusion, 16 out of those 18 patients showed an adequate hemodynamic response. The hemodynamic parameters at rest and during adenosine stress are shown on table 2. Two patients of the standard infusion group and one patient of the high-dose group developed transient advanced AV block. Significantly more patients complained of chest pain in the high-dose group (61% vs. 29%, p= 0.009) .On multivariate analysis, age > 65 years and ejection fraction <57% were the only independent predictors of blunted haemodynamic responsiveness to adenosine.

## Conclusions

A substantial number of patients do not show adequate peripheral hemodynamic response to standard-dose adenosine stress during perfusion CMR imaging. Age and reduced ejection fraction are predictors of inadequate response to standard dose adenosine. Our initial experience with a high-dose adenosine protocol (up to 210 mcg/kg/min) shows that it is well tolerated and results in adequate hemodynamic response in nearly all patients.

**Table 1:** Hemodynamic Parameters at Rest and During Adenosine Stress

**Standard dose Adenosine** (n=80)				**High dose Adenosine** (n= 18)			
	Rest	Peak	p-value	Rest	140μg/kg/min	Peak	p-value

HR(bpm)	70±13	96±16	<0.001	66±12	75±11	81±18*	0.011

SBP (mmHg)	137±18	139±19	0.21	139±23	134±24	132±22	0.68

DBP (mmHg)	80±12	81±12	0.55	82±12	79±11	77±14	0.57

RPP	9639±2399	13304±2781	<0.001	8975±1909	9945±1694	10672±2787	0.07

* p<0.05 vs. rest

DBP, diastolic blood pressure; HR, heart rate; RPP, rate pressure product; SBP, systolic blood pressure

